# A Uremic Goat Model Created by Subtotal Renal Artery Embolization and Gentamicin

**DOI:** 10.3390/biology10040292

**Published:** 2021-04-03

**Authors:** Maaike K. van Gelder, Joost C. de Vries, Sabbir Ahmed, Anneke S. Monninkhof, Gérard A. P. de Kort, Evert-Jan P. A. Vonken, Diënty H. M. Hazenbrink, Koen R. D. Vaessen, Tri Q. Nguyen, Marianne C. Verhaar, Jaap A. Joles, Karin G. F. Gerritsen

**Affiliations:** 1 Department of Nephrology and Hypertension, University Medical Center Utrecht, Heidelberglaan 100, 3584 CX Utrecht, The Netherlands; M.K.vangelder-7@umcutrecht.nl (M.K.v.G.); J.C.deVries-34@umcutrecht.nl (J.C.d.V.); a.s.monninkhof@gmail.com (A.S.M.); dienty.hazenbrink@embl.de (D.H.M.H.); M.C.Verhaar@umcutrecht.nl (M.C.V.); J.A.Joles@umcutrecht.nl (J.A.J.); 2 Division of Pharmacology, Utrecht Institute for Pharmaceutical Sciences, Utrecht University, Universiteitsweg 99, 3584 CG Utrecht, The Netherlands; s.ahmed@uu.nl; 3 Department of Radiology, University Medical Center Utrecht, Heidelberglaan 100, 3584 CX Utrecht, The Netherlands; G.A.P.deKort@umcutrecht.nl (G.A.P.d.K.); E.Vonken@umcutrecht.nl (E.-J.P.A.V.); 4 Central Laboratory Animal Research Facility, Utrecht University, Heidelberglaan 8, 3584 CS Utrecht, The Netherlands; K.R.D.Vaessen@uu.nl; 5 Department of Pathology, University Medical Center Utrecht, Heidelberglaan 100, 3584 CX Utrecht, The Netherlands; T.Q.Nguyen@umcutrecht.nl

**Keywords:** chronic kidney disease, models, animal, embolization, gentamicin, peritoneal dialysis, hemodialysis

## Abstract

**Simple Summary:**

To improve the treatment of patients with kidney disease, new renal replacement therapies are being developed. Prior to their use in humans with end-stage kidney disease (ESKD), these therapies need to be tested in animals with kidney disease. Goats seem particularly suitable because they are docile, have bodyweights comparable to humans and easily accessible neck veins to obtain blood access and tolerate frequent blood sampling, allowing for repeated monitoring. To obtain high blood concentrations of waste solutes that accumulate in patients with kidney disease and allow for the evaluation of solute removal by the new renal replacement therapies, we established kidney failure in five goats by partially blocking the blood supply of the kidneys via radiographic embolization. This resulted in stable moderate kidney disease. The administration of high-dose gentamicin caused a temporary further decline in kidney function. Although the response varied with animal, blood concentrations of waste solutes were representative of those found in ESKD patients. These animals can survive for more than ten months in good condition, allowing for the repetitive testing of new therapies in one animal, and therefore limiting the use of laboratory animals.

**Abstract:**

A large animal model of (end-stage) kidney disease (ESKD) is needed for the preclinical testing of novel renal replacement therapies. This study aimed to create stable uremia via subtotal renal artery embolization in goats and induce a temporary further decline in kidney function by administration of gentamicin. Renal artery embolization was performed in five Dutch white goats by infusing polyvinyl alcohol particles in branches of the renal artery, aiming for the embolization of ~80% of one kidney and complete embolization of the contralateral kidney. Gentamicin was administered to temporarily further increase the plasma concentrations of uremic toxins. After initial acute kidney injury, urea and creatinine plasma concentrations stabilized 1.5 ± 0.7 months post-embolization and remained elevated (12 ± 1.4 vs. 5.6 ± 0.8 mmol/L and 174 ± 45 vs. 65 ± 5.6 µmol/L, resp.) during follow-up (16 ± 6 months). Gentamicin induced temporary acute-on-chronic kidney injury with a variable increase in plasma concentrations of small solutes (urea 29 ± 15 mmol/L, creatinine 841 ± 584 µmol/L, phosphate 2.2 ± 0.3 mmol/L and potassium 5.0 ± 0.6 mmol/L) and protein-bound uremic toxins representative of patients with ESKD. A uremic goat model characterized by stable moderate uremia was established via subtotal renal artery embolization with the induction of temporary severe acute-on-chronic kidney injury by the administration of gentamicin, allowing preclinical in vivo validation of novel renal replacement technologies.

## 1. Introduction

Novel renal replacement therapies (RRT) are under development to improve the treatment of patients with end-stage kidney disease (ESKD). These innovations include miniature dialysis machines for hemodialysis (HD) and peritoneal dialysis (PD), a bioartificial kidney and innovative dialysis membranes [1,2,3]. Prior to clinical evaluation in humans, the safety and efficacy of these therapies must be evaluated in a uremic large-animal model. Although small animal models of chronic kidney disease (CKD) are well-established, these are not suitable for testing “full scale” dialysis devices because of their small size, which does not match the volume of the extracorporeal circuit in humans and limits the maximum collection volume of blood. The ideal animal model for testing renal replacement therapies has plasma concentrations of uremic toxins representative for patients with ESKD, without the need for maintenance dialysis therapy. However, uremia in the existing large-animal models is either too mild, prohibiting an adequate evaluation of device efficacy [4,5], or too severe, resulting in impractical dialysis dependency and limited survival [6,7,8,9], both of which are also undesirable for animal welfare considerations. In addition, few studies have performed PD in a large-animal model and peritoneal membrane transport characteristics were never reported [8,10]. Hence, it is unknown whether these animal models are representative of PD patients.

The aim of the present study was to create a stable model of CKD in goats by performing a subtotal nephrectomy via transfemoral renal artery embolization. This method allows for the selective embolization of branches of the renal artery while sparing others, and is minimally invasive. Gentamicin, a drug with well-known nephrotoxicity, was administered to temporarily increase plasma concentrations of uremic toxins on demand, in order to study their removal during dialysis. Both HD and PD experiments were performed as proof of concept.

## 2. Materials and Methods

### 2.1. Experimental Enimal

The study was approved by the Animal Experiments Committee (Utrecht, The Netherlands) and performed in accordance with national guidelines for the care and handling of animals. Clinically healthy female Dutch White adult goats (capra aegagrus hircus) (*n* = 5) weighing 50–80 kg were selected, because these animals are docile, have easily accessible neck veins for placement of a central venous catheter and bodyweights (and distribution volumes) comparable to humans [11]. Goats were purchased from V.O.F. de Römer (Heythuysen, The Netherlands). Before the start of the experiment, clinical-chemistry and hematology screening tests were performed and goats were screened for caprine arthritis encephalitis, caseous Lymphadenitis, paratuberculosis, and bovine virus diarrhea, and vaccinated against Q-fever. Goats were housed individually in indoor stables following embolization to allow the femoral catheter insertion site to heal and, when instrumented with a central venous catheter or abdominal PD catheter, to prevent catheter dislodgement by other goats. Otherwise, goats were housed in groups. The temperature of the animal room was maintained between 18 and 21 °C and artificial lighting was provided. Goats were offered 300 g of dry feed (Kasper Faunafood, Woerden, The Netherlands) per day. Hay and water were provided ad libitum.

### 2.2. Subtotal Renal Artery Embolization

Subtotal renal artery embolization was established in two stages. First, partial embolization of one kidney was performed, followed by complete embolization of the contralateral kidney, aiming to preserve ~15% of total kidney tissue, based on previous studies that showed that preserving 8–10% [7] and 17–25% [5,6] of the kidney resulted in a uremic state that was either too severe or too mild, respectively. On the day of the embolization, goats received detomidine 0.04 mg/kg, followed by the administration of propofol 2 mg/kg i.v. for the induction of general anesthesia and propofol 10 mg/kg/h and remifentanil 0.03 mg/kg/h for the maintenance of anesthesia. Analgesic medication was administered postoperatively (Metacam 0.5 mg/kg per day i.v. for 5 days, starting on the day before the procedure; BuTrans band aid, placed the day prior to surgery for 6 days). A single dose of antibiotics (amoxicillin/clavulanic acid) was administered to prevent infection. A 5 French sheath was placed in the femoral artery using Seldinger technique. A Glidecath™ catheter (Terumo Cobra 2, 5 Fr, REF: RF-XB55108M, Leuven, Belgium) was used as a guiding catheter for a micro-catheter system (Terumo Progreat, 2.7 Fr, REF: MC-PP27131, Leuven, Belgium). The tip of the catheter was advanced into the renal artery (complete embolization) or segmental and interlobar arteries (partial embolization) and polyvinyl alcohol (PVA) embolization particles (Contour™, Embolization Particles, 45–150 microns, Boston Scientific, Marlborough, Massachusetts, MA, USA) were released into branches of the renal arteries using fluoroscopy, followed by flushing with physiologic saline. The remaining area of perfused kidney tissue was visualized by contrast angiography.

### 2.3. Measurement of Glomerular Filtration Rate and Renal Plasma Flow

Glomerular filtration rate (GFR, mL/min) and effective renal blood flow (ERBF, mL/min) were measured by inulin and para-amminohippuric acid (PAH) clearances (Formulas 1 and 2), respectively, before and 1.5 ± 0.7 months after embolization, once urea and creatinine plasma concentrations had stabilized. Because the production of inulin by Fresenius Kabi was discontinued during the study, GFR was determined by iohexol clearance in two goats. Two venous lines were placed, one for blood sampling and one for inulin (or iohexol) and PAH infusion. An intravenous bolus of inulin 22.5 mg/kg (Sinistrin, Inutest^®^ 25%, Fresenius Kabi, Graz, Austria) and PAH 6 mg/kg (Sigma-Aldrich, Saint Louis, Missouri, MO, USA) dissolved in sodium chloride 0.9% (Sigma-Aldrich, Saint Louis, Missouri) was administered, followed by continuous inulin (11.25 mg/min) and PAH (11 mg/min) infusion. Alternatively, a single intravenous bolus of iohexol 5 mL (Omnipaque 300 mg/mL, GE Healthcare, Chigago, Illinois, IL, USA) was administered. Venous blood samples were collected before start and after 10, 60, 120, 180, 210, 240, 270 and 300 min, to measure inulin (or iohexol), PAH and hematocrit. Steady state plasma concentrations were used for the calculation of plasma clearance

(1) $$\mathrm{Cl}\;\left(\mathrm{mL}/\min\right)=\frac{\mathrm{Dose}\;\left(\mathrm{mg}/\min\right)}{\left(\mathrm{Cp}1+\mathrm{Cp}2\right)/2}$$

where Cl = plasma clearance, and Cp = the mean plasma concentration of two consecutive measurements (Cp1 and Cp2).

(2) $$\mathrm{ERBF}\;\left(\mathrm{mL}/\min\right)=\frac{\mathrm{Cl}\;\mathrm{PAH}\;\left(\mathrm{mL}/\min\right)}{1\;-\;\mathrm{hematocrit}}$$

where Cl PAH = plasma clearance of para-ammino hippuric acid, and ERBF = effective renal blood flow.

### 2.4. The Effect of Gentamicin on Uremic Toxins

Gentamicin 5–10 mg/kg was administered twice daily for 4–11 days intramuscularly in the gluteus maximus to measure the effect on plasma concentrations of urea, creatinine, phosphate and potassium, all measured as described [11], cystatin C (13.3 kDa), and the following protein-bound uremic toxins (PBUTs): indoxyl sulfate, indole-3-acetic acid, kynurenine, kynurenic acid, p-cresyl glucuronide, p-cresyl sulfate and hippuric acid. Cystatin C was measured with an Atellica Neph 630 analyser (Siemens, München, Germany) using an immunoassay. Of note, we tried to measure β2-microglobulin (12.8 kDa) which is commonly used as a marker of middle molecules, using a commercially available ELISA kit (catalog number: MB737860, mybiosource, San Diego, CA, USA), but did not succeed in reliably determining β2-microglobulin in goat serum. To measure total PBUT plasma concentrations, plasma samples were diluted and deproteinized by acetonitrile and analyzed centrally using ultrahigh-performance liquid chromatography-mass spectroscopy (UHPLC-MS) analysis [12]. PBUT concentrations were quantified based on the peak area ratio of the sample and internal standard. In case of concentrations at the lower end of the quantifiable concentration range, weighted regression of 1/y^2^ was applied to obtain a more accurate concentration.

### 2.5. Biochemical Monitoring

Venous blood sampling was performed by puncture of the external jugular vein pre- and post-embolization until kidney function stabilized and blood was taken at least once per month to monitor plasma concentrations of urea, creatinine, phosphate, potassium and hemoglobin (g/L).

### 2.6. Blood Pressure

Arterial blood pressure was measured two months after embolization via cannulation of the a. auricularis in awake goats in standing position, and was compared to blood pressure in healthy goats.

### 2.7. Acetazolamide and Ramipril

An important aspect of dialysis treatment is the release of bicarbonate (or equivalent) to neutralize the daily non-volatile acid production that is not sufficiently excreted by the kidneys in ESKD, and therefore limit metabolic acidosis. To study bicarbonate release during treatment, which depends on a transmembrane concentration gradient across the dialysis or peritoneal membrane, the plasma bicarbonate concentration of the experimental animal must be representative for patients with ESKD. Because plasma bicarbonate concentrations in goats were relatively high (28.9 ± 3.1 mmol/L) compared with maintenance HD patients (63% of HD patients have a plasma bicarbonate concentration below 23 mmol/L [13]), a combination of acetazolamide 500 mg once daily and ramipril 1.25 mg once daily was administered orally for 3–10 days to lower plasma bicarbonate concentrations. Acetazolamide, a carbonic anhydrase inhibitor, increases renal bicarbonate excretion [14]. Ramipril, an angiotensin converting enzyme inhibitor, also compromises renal acid excretion [14].

Venous blood samples were drawn before the start and once daily prior to medication administration in a lithium–heparin tube with gel-separator. The tubes were centrifuged immediately at 4 °C and left unopened until analysis. Analysis was performed within 2 h to assure bicarbonate stability [15].

### 2.8. Complement Activity

The complement system is a group of proteins that, when activated, cause cell lysis and facilitate phagocytosis through opsonisation. A hemolytic assay was used to test the functional capability of the classical (CH50) and alternative (AP50) complement pathway in the serum of uremic goats to lyse human erythrocytes pre-coated with rabbit anti-human antibodies, as described by Moreno-Indias et al. [16]. Blood (*n* = three samples) was collected from two female white goats in clot-activating tubes (CAT, BD vacutainer) and centrifuged at 2130 g for 10 min. Serum was separated and centrifuged again at 2500 g for 10 min. All reagents and samples were stored on ice during the assay to prevent the spontaneous activation of complement proteins. Sensitized human erythrocytes were prepared from the blood of healthy volunteers (EDTA tube, BD vacutainer). The tube was centrifuged at 2130 g for 10 min, plasma was discarded and cells were washed with PBS-0.5 mmol/L EDTA and divided over two separate tubes. Cells were washed twice more with the assay-specific buffers (DGHB++ buffer was used for CH50 assay and DGHB-Mg-EGTA for the AP50 assay), which were prepared as described [16]. The concentration of human erythrocytes was adjusted to 10^8^ erythrocytes/mL. Subsequently, human erythrocytes were sensitized with rabbit anti-human erythrocyte membrane (1.1 mg/mL, Fitzgerald, Acton, MA, USA) at a concentration of 100 µL in 10 mL of 10^8^ erythrocytes/mL and incubated for 30 min at 56 °C. Cells were washed twice and the concentration was readjusted to 10^8^ erythrocytes/mL. CH50 and AP50 assays were performed simultaneously in a Masterblock 96-well plate (Greiner bio-one, Alphen aan den Rijn, the Netherlands). An initial 2-fold dilution was performed, starting with 200 µL of goat serum (in triplo), followed by serial 10-fold dilutions with DGHB++ or DGHB-Mg-EGTA buffer, respectively. In addition, a blank well containing only buffer (no lysis) and a well containing distilled water (total lysis) were included. A 200-µL sample of sensitized human erythrocytes was added to each well, followed by incubation in a water bath at 37 °C for 60 min. After incubation, a sample was taken from each well, centrifuged for 10 min at 2500 g and the degree of hemolysis was quantified by measuring the absorbance of the released haemoglobin into the supernatant at 405 nm using a spectrophotometer (Multiskan FC Microplate Photometer type 357). Complement-dependent lysis of erythrocytes was calculated as the percentage lysis relative to cells lysed in water (100% lysis) and cells incubated in buffer (0% lysis). Percentage lysis was plotted against serum dilution. CH50 and AP50 activity was expressed as the amount of goat serum necessary to cause 50% lysis.

### 2.9. Histopathology

After euthanasia, via administration of pentobarbital 150 mg/kg, tissue samples were taken from embolized and non-embolized kidney tissue, fixated in 10% formalin and embedded in paraffin. Periodic acid-schiff staining was performed on deparaffinized 4-µm cross-sections.

### 2.10. Hemodialysis Experiments

In total, eight 4-h HD sessions were performed in one goat to treat acute-on-chronic kidney injury (three session in five days for gentamicin-induced AKI and five sessions in nine days prior to euthanasia). Prior to dialysis, a central venous catheter (CVC, Covidien Palindrome Chronic Dual Lumen Catheter 14.5 Fr/Ch (4.8 mm) × 55 cm −3.1 mL V3.1 mL) was inserted into the external jugular vein under anesthesia with detomidine and propofol as described [11]. This relatively long CVC (55 cm) was required to achieve correct positioning of the catheter tip in the right atrium, or just above in the superior vena cava. A conventional HD machine (Nikkiso DBB-05) and F8HPS (Fresenius Medical Care) low-flux dialyzer were used. Dialysate was prepared using a reverse osmosis water purification system, acid concentrate D831 (Dirinco, Oss, The Netherlands) and BiCart (Gambro, Lund, Sweden). A blood and dialysate flow rate of 400 and 500 mL/min, respectively, were applied. Anticoagulation was achieved by an initial loading dose of unfractionated heparin (10.000 IU), followed by constant infusion (3.500 IU/h) to maintain activated clotting time > 500 s. Venous blood samples were drawn from the CVC before and after treatment to calculate reduction ratios (RR, %) for urea, creatinine and phosphate and equilibrated Kt/V (eKt/V) urea, according to the second-generation Daugirdas formula [17]. After dialysis, the CVC was locked with 10 mL heparinized (500 IU/mL) glycerol 50% and flushed daily with NaCl 0.9%. Enoxaparin 75 mg s.c. and amoxicillin/clavulanic acid 10/1 mg/kg i.v. were administered once daily to prevent line thrombosis and infection, respectively.

### 2.11. Standard Peritoneal Permeability Analysis

Two goats were instrumented with a straight Tenckhoff PD catheter (Argyle, Covidien, Tenckhoff, 1 cuff, 47 cm) under general anesthesia. The catheter was tunneled to the back to prevent catheter manipulation and flushed daily with 50 mL NaCl 0.9%. After 379–382 days (goat 1) and after 4 days (goat 2), a standard peritoneal permeability analysis (SPA) was performed to assess peritoneal membrane transport status in terms of dialysate to plasma ratio (D/P) creatinine and the mass transfer area coefficient (MTAC) of urea and creatinine, as described by Pannekeet et al. [18]. In brief, a filling with 2-L of Physioneal 35 1.36% glucose (Baxter) was performed and PD effluent samples were collected from the peritoneal catheter after 10, 20, 30, 60, 120, 180 and 240 min to measure urea and creatinine concentrations. Residual intraperitoneal volume was determined before and after the SPA dwell, based on the dilution of total protein concentration after rinsing of the peritoneal cavity with fresh Physioneal. A venous blood sample was drawn before and after the SPA to measure plasma urea, creatinine and phosphate concentrations. The MTAC was calculated according to the modified Waniewski model [19]. The PD catheter was removed after two months.

### 2.12. Statistical Analysis

Descriptive statistics are reported as mean ± SD or median (interquartile range), as appropriate. Linear interpolation was used for missing plasma concentrations of uremic toxins during follow-up. Comparison between two repeated measurements during follow-up were performed using paired *t*-tests or Wilcoxon signed rank test, as appropriate. A *p* value < 0.05 was considered significant. Analyses were performed using IBM SPSS Statistics 25 (SPSS Inc., Chicago, IL, USA) and Graphpad Prism (Version 7.04, San Diego, California, CA, USA).

## 3. Results

### 3.1. Subtotal Renal Artery Embolization

**Figure 1:**
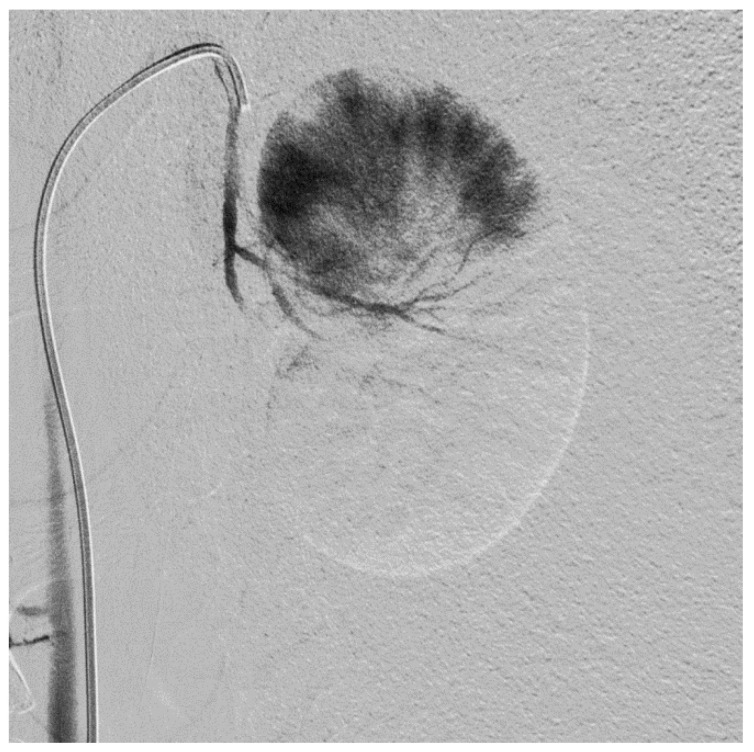
Embolization of the caudal pole of the left kidney. Perfusion of blood vessels in the cranial pole of the left kidney is visualized using contrast angiography.

Subtotal renal artery embolization was performed in five goats. In the first goat, subtotal nephrectomy of ~83% of total kidney tissue was performed in two stages. First, partial embolization of one kidney (~67%. Figure 1) was performed to allow for hypertrophic adaptation of the remnant kidney and prevent dialysis dependency after complete embolization of the contralateral kidney 35 days later. This approach resulted in mild uremia. Plasma urea and creatinine concentrations increased from to 4.5 mmol/L to 12.3 mmol/L and 64 µmol/L to 164 µmol/L, pre- versus post-embolization, respectively, and phosphate plasma concentrations decreased from 1.73 mmol/L to 1.44 mmol/L, respectively. Therefore, in subsequent goats, the embolization area was increased to ~90% of total kidney tissue and the partial and complete embolization were performed in one procedure. This approach resulted in higher peak urea, creatinine and phosphate plasma concentrations immediately post-embolization, but similar plasma concentrations after stabilization of kidney function at three months post-embolization (12 ± 1.6 mmol/L, 168 ± 15 µmol/L (Figure S1) and 1.45 ± 0.28 mmol/L, respectively (Table 1)). GFR decreased with 62% and ERBF with 71% after 1.5 ± 0.7 months following embolization.

Postoperative clinical recovery of goats was rapid, and no serious adverse events occurred. After 24 h, temporary signs of mild to moderate pain (teeth grinding, abnormal posture) were observed, which responded well to additional analgesic medication. One goat developed a groin hematoma post-operatively, located at the femoral catheter insertion site, which resolved spontaneously.

### 3.2. Survival, General Clinical Condition and Bodyweight

Mean follow-up time was 16 ± 6 months. Renal artery embolization resulted in stable moderately elevated plasma concentrations of urea (12 ± 1.4 vs. 5.6 ± 0.8 mmol/L) and creatinine (174 ± 45 vs. 65 ± 5.6 µmol/L), without the need for maintenance dialysis (Figure S1). Apart from the post-operative period and prior to euthanasia, no clinical signs of severe discomfort (e.g., tooth grinding and abnormal posture) were observed. In general, bodyweight was stable or increased during follow-up. A temporary decline in bodyweight of 2.3 ± 3.7 kg was observed during acute-on-chronic kidney injury following gentamicin administration, which increased after recovery of kidney function. Hemoglobin concentrations decreased in all goats within 1–2 months and were responsive to darbepoietin (Aranesp 100 µg once every 2–3 months i.m.) and iron therapy (Alfafer 10%, 600 mg iron and 180 µg cyanocobalamine once every 2–3 months i.m.).

Two goats were euthanized after 15 and 27 months of follow-up because of unexplained severe acute-on-chronic kidney injury, which was not induced by gentamicin administration, and did not recover spontaneously. The first goat received one course of gentamicin (10 mg/kg twice daily for 7 days) at 8 months of follow-up, which caused severe acute-on-chronic kidney (requiring temporary HD) with partial recovery to stable severe uremia (Figure S2A). The second goat received six gentamicin courses (5–10 mg/kg twice daily for 4–8 days) up to 23 months of follow-up, which resulted in moderate increases in plasma toxin concentrations and (partial) recovery to stable moderate uremia (eGFR 30–40 mL/min, Figure S2B). One goat was euthanized after 10 months because of acute-on-chronic kidney injury following gentamicin administration, which was augmented by dehydration which could not be treated by intravenous fluids due to an inability to obtain venous access. The two remaining goats were euthanatized after 16 months because of an unrelated infection (*n* = 1) and because of an intra-abdominal mass suspect for a liver cyst or abscess with signs of severe liver failure (*n* = 1).

### 3.3. Effect of Gentamicin on Plasma Urea, Creatinine, Phosphate, Potassium Plasma Concentrations

**Figure 2:**
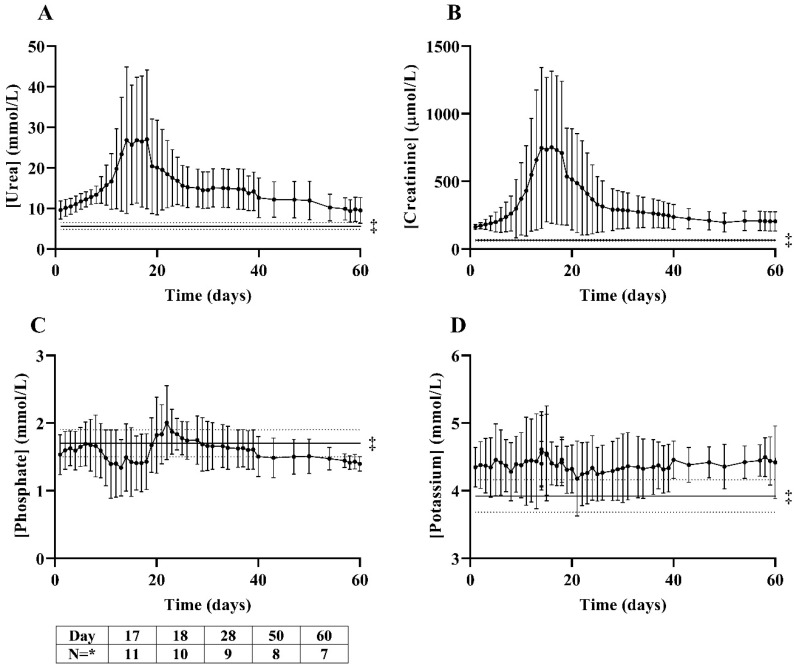
Mean (±SD) plasma concentrations of urea (mmol/L, (**A**)), creatinine (µmol/L, (**B**)), phosphate (mmol/L, (**C**)) and potassium (mmol/L, (**D**)) after administration of gentamicin 10 mg/kg twice daily for 7–10 days in goats (*n* = 11 courses in *n* = 5 goats, 1–3 gentamicin courses per goat). Gentamicin was started at day 1. * Number of gentamicin courses per timepoint. ‡ Mean (±SD) pre-embolization plasma concentration.

In total, nineteen gentamicin courses (5–10 mg/kg twice daily for 4–11 days) were administered to five goats (*n* = 1–8 per goat). The administration of gentamicin 5–10 mg/kg twice daily for <7 days appeared to be insufficient to consistently induce severe acute-on-chronic kidney injury (Table S1). The administration of gentamicin 10 mg/kg twice daily for 7–10 days caused a transient increase in plasma concentrations of urea and creatinine from 9.5 ± 2.2 mmol/L to 29.4 ± 15.4 mmol/L (Figure 2A) and 164 ± 20 µmol/L to 841 ± 584 µmol/L (Figure 2B), respectively, after 16 ± 2 days. Creatinine plasma concentrations were higher than 250 µmol/L from day 10 ± 2 for a duration of 19 ± 9 days (excluding one goat in whom plasma creatinine concentration stabilized at ~350 µmol/L during six months of follow-up until death after severe gentamicin-induced acute-on-chronic kidney injury requiring temporary HD (Figure S2A)). In one goat, gentamicin 10 mg/kg for 7–8 days only resulted in a very moderate increase in plasma urea and creatinine concentrations (Table S1), which was ascribed to substantial residual renal function. Repeated exposure of the same goat to gentamicin 10 mg/kg twice daily for 7–10 days did increase plasma solute concentrations. In two goats, a severe decline in kidney function was observed following administration of gentamicin 10 mg/kg for 7 days, which necessitated intermittent HD (three sessions in five days) in one goat (Figure S2A, as mentioned above), while in the other goat, no vascular access could be obtained, and euthanasia was performed to prevent severe animal discomfort (see above). Of note, the latter goat had received gentamicin twice before and had just recovered from a severe episode of gentamicin-induced acute injury one month earlier. Severe acute-on-chronic kidney injury due to gentamicin was often accompanied by dehydration requiring intravenous fluid suppletion.

Administration of gentamicin 10 mg/kg twice daily for 7–10 days caused an initial decrease in phosphate plasma concentrations from 1.54 ± 0.29 mmol/L to 1.09 ± 0.38 mmol/L after 13 ± 3 days, after which plasma concentrations increased to 2.18 ± 0.28 mmol/L after 20 ± 5 days. Phosphate plasma concentrations were higher than 1.5 mmol/L from day 14 ± 7 for a duration of 10 ± 6 days. Potassium plasma concentrations increased from 4.3 ± 0.3 mmol/L to 5.0 ± 0.6 mmol/L.

### 3.4. Protein-Bound Uremic Toxins and Cystatin C Plasma Concentrations

**Figure 3:**
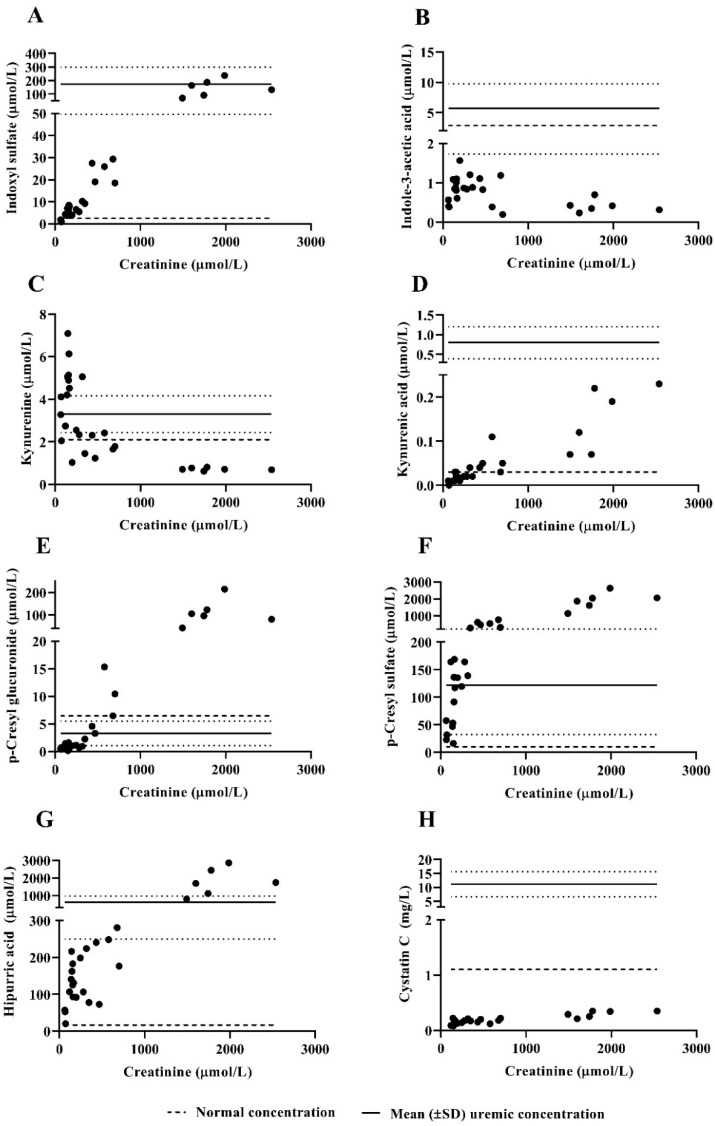
Plasma concentrations of indoxyl sulfate (µmol/L, (**A**)), indole-3-acetic acid (µmol/L, (**B**)), kynurenine (µmol/L, (**C**)), kynurenic acid (µmol/L, (**D**)), p-cresyl glucuronide (µmol/L, (**E**)), p-cresyl sulfate (µmol/L, (**F**)), hippuric acid (µmol/L, (**G**)) and cystatin C (mg/L, (**H**)) in *n* = 5 goats were measured at varying levels of uremia as represented by increasing creatinine plasma concentrations (µmol/L) following embolization, gentamicin administration or non-gentamicin induced acute-kidney injury. Each datapoint represents a plasma concentration of an individual goat during follow-up. Mean (±SD) (solid line with dotted lines showing SD) uremic plasma concentration and normal plasma concentration (dashed line) in humans are based on the literature [20,21].

At higher creatinine plasma concentrations during acute-on-chronic kidney injury (following gentamicin administration or prior to termination), plasma concentrations of indoxyl sulfate, p-cresyl glucuronide, p-cresyl sulfate and hippuric acid were within the range (indoxyl sulfate, p-cresyl sulfate, hippuric acid) or higher (p-cresyl sulfate) than uremic plasma concentrations (Figure 3, Table 1) [20,21]. Kynurenic acid only showed a limited increase to levels just above those in healthy subjects. In contrast, plasma concentrations of kynurenine were relatively high at lower creatinine plasma concentrations. No association was observed between indole-3-acetic acid or cystatin C and creatinine plasma concentrations.

### 3.5. Blood Pressure

Systolic blood pressure measured two months post-embolization was not different compared with blood pressure in healthy goats (117 ± 5 mmHg vs. 120 ± 13 mmHg, respectively, *p* = 0.16) and diastolic blood pressure tended to be lower (73 ± 9 mmHg vs. 84 ± 12 mmHg, respectively, *p* = 0.06).

### 3.6. Effect of Acetazolamide and Ramipril on Plasma Bicarbonate Concentrations

**Figure 4:**
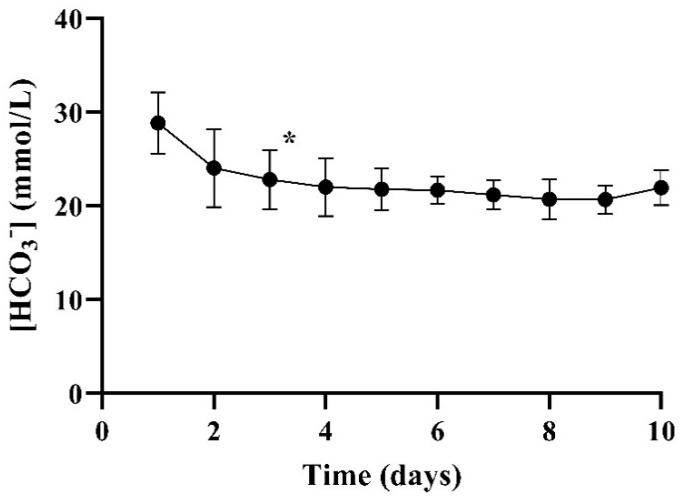
Mean (±SD) plasma bicarbonate concentrations after oral administration of acetazolamide 500 mg/day and ramipril 1.25 mg/day for 3–10 days (*n* = 10 courses in 3 goats, 1–6 courses per goat) is presented. * Day 1 vs. 3: *p* < 0.001 (paired *t*-test).

Treatment with acetazolamide 500 mg/day and ramipril 1.25 mg/day for three days caused a decrease in plasma bicarbonate concentrations from 28.9 ± 3.1 mmol/L to 20.3 ± 0.9 mmol/L (*p* < 0.001) (Figure 4). Continuing administration beyond three days did not cause a further decrease in plasma bicarbonate concentrations.

### 3.7. Complement Activity

Activity of the classical (CH50) and alternative (AP50) complement pathway was determined during stable uremia (i.e., not during gentamicin-induced acute kidney injury) using a haemolytic assay (Figure S3). For both the CH50 and AP50 assay, an 8-fold dilution of the goat serum resulted in 50% lysis.

### 3.8. Histopathology

**Figure 5:**
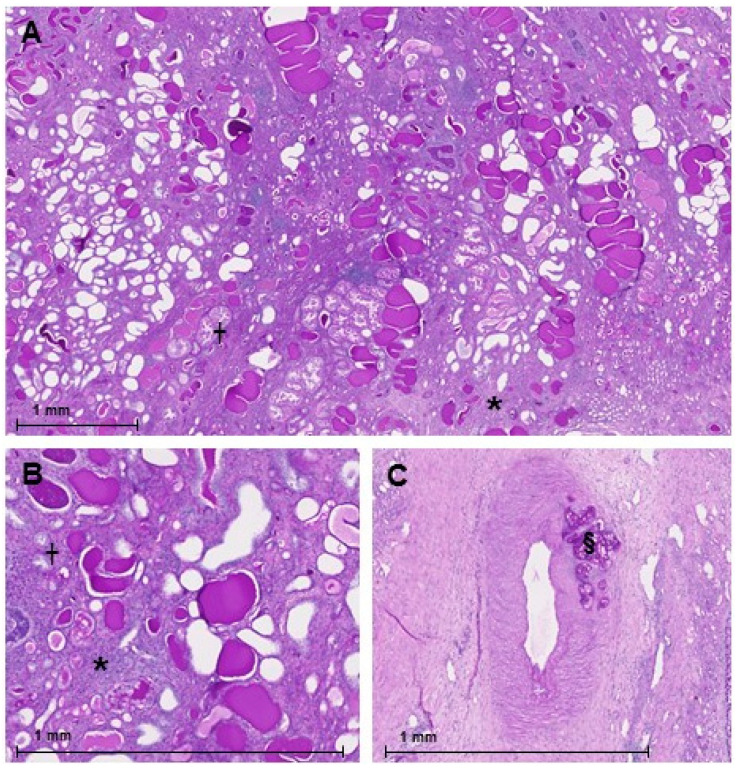
Periodic Acid Schiff staining of a partially embolized kidney. (**A**,**B**): areas with severe interstitial fibrosis (*) and tubular atrophy (†) are present in the non-embolized part of the kidney. (**C**): interlobar artery with an embolus containing polyvinyl alcohol particles that completely occludes a side branch (§).

Representative kidney tissue sections of a goat that was euthanized after 16 months are presented in Figure 5. The deposition of embolization particles is visible in the capillary bed (Figure 5C). In the non-embolized part of the partially embolized kidney, severe interstitial fibrosis and tubular atrophy is visible (Figure 5A,B).

### 3.9. Hemodialysis Experiments

In total, eight 4-h HD sessions were performed in one goat to treat acute-on-chronic kidney injury (three sessions in five days for gentamicin-induced AKI and five sessions in nine days prior to euthanasia). The insertion of a CVC and the dialysis sessions were uncomplicated. Mean RRs of urea, creatinine and phosphate were 67 ± 5.2%, 66 ± 5.5% and 52 ± 2.2%, respectively, and eKt/V urea was 1.1 ± 0.04 per session. The CVC’s remained patent up to one month after which they were removed because HD was no longer required.

### 3.10. Standard Peritoneal Permeability Analysis (SPA)

Placement of the peritoneal catheter was uncomplicated. Peritoneal membrane transport characteristics, MTAC and D/P creatinine were low (Table 2), corresponding with a low peritoneal transport status (i.e., D/P creatinine at 4 h <0.55 [22]). The catheter was removed after two months. During the two-month period with the peritoneal catheter in situ, there were no signs of exit site or systemic infection or functional catheter-related problems.

## 4. Discussion

In the present study, a uremic goat model was established via subtotal renal artery embolization with the induction of temporary severe acute-on-chronic kidney injury by the administration of gentamicin.

Adult white goats (Capra aegagrus hircus) were selected because they are particularly suitable for the evaluation of novel HD technologies, because of their long neck, which facilitates the placement of a central venous catheter [11]. In addition, body weight and the distribution volume of urea are comparable to humans [23].

Renal artery embolization resulted in stable moderately elevated plasma concentrations of urea and creatinine without the need for maintenance dialysis. This minimally invasive procedure was characterized by a short recovery time, reducing animal discomfort compared with open surgical techniques. Gentamicin induced temporary acute-on-chronic kidney injury on demand, with near complete recovery of kidney function to pre-gentamicin levels. We observed a more severe decline in kidney function after repeated exposure to high doses or prolonged therapy. In addition, the response varied per animal, which may be due to differences in residual kidney function [24,25,26]. We therefore recommend the careful monitoring of kidney function during gentamicin administration, and discontinuation of the drug once creatinine plasma concentration starts to increase, as kidney function will continue to decline after the discontinuation of gentamicin administration as a result of drug accumulation in proximal tubular cells. In contrast to uremic animal models with severe kidney failure [6,7,8,9], the prolonged survival of our animals allowed for repeated testing in the same animal, limiting the number of laboratory animals. In addition, our CKD model is suitable for studies requiring long-term follow-up. However, despite having stable uremia for more than a year, acute-on-chronic kidney injury developed in two animals 15 and 27 months after embolization, which was not induced by gentamicin. Our histopathological data indicate that this may have been caused by hyperfiltration-induced injury combined with tubular injury and renal fibrosis in the remnant kidney, resulting in a progressive decline in kidney function [27].

The primary mechanism of gentamicin-induced nephrotoxicity is tubular necrosis. The drug is excreted by glomerular filtration and partially reabsorbed by the proximal tubular cells, resulting in tubular cell damage and necrosis, followed by cellular regeneration [28]. Glomerular filtration may also decrease due to intratubular obstruction and gentamicin-induced activation of the renin-angiotensin system, resulting in local vasoconstriction. Accordingly, we initially observed a decrease in phosphate plasma concentrations, presumably due to tubular injury-induced impaired renal phosphate reabsorption [29], followed by an increase in phosphate plasma concentrations as a result of a decline in glomerular filtration rate.

In the ideal uremic large-animal model, all three classes of uremic toxins (small water-soluble solute (<500 Da), middle molecules (≥500 Da) and PBUTs) should be quantifiable, with plasma concentrations representative of patients with kidney disease. Gentamicin-induced acute-on-chronic kidney injury resulted in a considerable increase in plasma concentrations of small water-soluble uremic solutes (urea, creatinine and phosphate) to levels comparable to plasma concentrations in ESKD patients [30,31]. Considering that creatinine plasma concentrations were elevated > 250 µmol/L between day 10–29 and phosphate plasma concentrations were elevated > 1.5 mmol/L between day 14–24 following gentamicin administration, the ideal test interval for experiments requiring more severe uremia (e.g., HD experiments) is between day 14 and 29. The increase in PBUT plasma concentrations was variable. Whereas plasma concentrations of indoxyl sulfate, p-cresyl glucuronide, p-cresyl sulfate and hippuric acid were comparable to uremic plasma concentrations in humans [20,21], plasma concentrations of indole-3-acetic acid and kynurenine were relatively low or decreased at higher creatinine plasma concentrations. This might reflect differences in colonic microbial metabolism between goats and humans and reduced dietary protein intake caused by uremic anorexia [12]. Finding a suitable middle molecule was challenging. Since beta-2 microglobulin (11.8 kDa), which is commonly used as a middle-molecular marker, could not be quantified in goats, we measured cystatin C (13 kDa). Although plasma concentrations of cystatin C in goats were lower than in humans, this middle molecule can be quantified, and thus can be used to determine middle molecule removal by novel renal replacement therapies (e.g., by measuring cystatin C concentrations up- and downstream of the dialyzer).

Blood pressure was not increased in our model two months after embolization. Similarly, Misra et al. found that after an initial phase of hypertension post-surgery, blood pressure had returned to baseline values after two weeks in a uremic swine model induced by renal artery embolization, sparing ~25% of total kidney tissue [5]. Chade et al. did observe a sustained increase in blood pressure in a uremic swine model induced by bilateral renal artery stenosis [4]. Possibly, this method caused a more significant activation of the renin–angiotensin system. Because blood pressure measurements via auricular artery cannulation merely provide an occasional measurement, (continuous) telemetric blood pressure registration can be used for continuous long-term blood pressure monitoring [32].

Plasma bicarbonate levels were relatively high in goats as compared to humans, and could be reduced to levels representative of patients with ESKD [13]) by the administration of acetazolamide and ramipril for three days. This allows for the evaluation of acid-base balance, including bicarbonate release to the patient, during treatment with novel renal replacement therapies.

For medical devices that are in direct contact with circulating blood, such as HD machines, testing of complement activation is recommended by the International Organization for Standardization (ISO) 10993 standard 10993 “Biological evaluation of medical devices—Part 4” [33] and FDA [34]. Usually, complement activation is assessed in vitro (e.g., by measuring SC5b-9 fragment activation using an ELISA), but these assays fail to simulate the complex interactions of various chemical and physical properties of the device with the complement system in vivo during the treatment of patients [33,34]. Therefore, measuring complement in vivo could help to assess complement activation under conditions more representative for intended use in humans. We found that complement activation via the classical and alternative pathway could be measured using the hemolytic assay as described [16]. As we only tested during stable uremia without clinical signs of complement activation (i.e., inflammation), future studies must evaluate whether this hemolytic assay can be used to detect enhanced complement activation, e.g., for comparison of complement activation during exposure to novel renal replacement therapies and conventional treatment.

HD was successfully performed in the uremic goats. Urea reduction ratios (URR) were comparable to URR in humans [35] and no catheter-related complications occurred. For PD, the uremic goat model may be less representative for the human situation because peritoneal transport status was relatively low in the goats compared with PD patients. In comparison, only 5% of PD patients has a low transport status (67% has high-average to high peritoneal transport status) [36]. MTACs of urea, creatinine and phosphate were ~2.7-fold, ~1.9-fold and ~1.5-fold lower than in PD patients [37,38,39]. Peritoneal transport usually increases with treatment time due to neoangiogenesis caused by prolonged exposure to glucose-containing PD solutions [40], resulting in an increase of the effective membrane surface area. However, despite their low absolute peritoneal mass transport of solutes, uremic goats could be used for comparison of uremic toxin removal between an investigational PD treatment and a conventional static dwell.

This study has several limitations. First, a single intra-arterial blood pressure measurement was performed two months after embolization to evaluate blood pressure during CKD as continuous telemetric blood pressure monitoring was not available. However, for the purpose of monitoring blood pressure during HD, we demonstrated that the placement of a temporary intra-arterial catheter in the auricular artery is technically feasible in goats [41]. Second, the number of animals in the present study is relatively small compared with that used in previous studies for the development of uremic large-animal models (7–26 animals). Because our model allows for prolonged follow-up, we could demonstrate the feasibility of subtotal renal artery embolization and evaluate the response to gentamicin administration in a limited number of goats.

## 5. Conclusions

In conclusion, a uremic goat model characterized by stable moderate uremia was established via subtotal renal artery embolization with the induction of temporary severe acute-on-chronic kidney injury by administration of gentamicin allowing preclinical in vivo validation of novel dialysis technologies. The extent to which gentamicin increased uremic toxin plasma concentrations varied and may depend on residual kidney function. The animals can be maintained in good clinical condition for more than ten months without the need for maintenance dialysis, with on-demand aggravation of kidney failure if required, thus limiting the use of laboratory animals.

## Figures and Tables

**Table 1:** The effect of embolization and gentamicin on plasma concentrations of urea, creatinine and phosphate and on glomerular filtration rate and effective renal blood flow.

Measurement	Pre-Embolization	Post-Embolization	AKI
Urea (mmol/L)	5.6 ± 0.8	12 ± 1.4	29.4 ± 15.4
Creatinine (µmol/L)	65 ± 5.6	167 ± 13	841 ± 584
Phosphate (mmol/L)	1.73 ± 0.19	1.44 ± 0.24	2.18 ± 0.28
Indoxyl sulfate (µmol/L)	1.39 ± 0.50		69 ± 76
Indole-3-acetic acid (µmol/L)	0.46 ± 0.10		0.67 ± 0.35
Kynurenine (µmol/L)	3.15 ± 1.04		1.67 ± 1.18
Kynurenic acid (µmol/L)	0.01 ± 0.01		0.09 ± 0.07
p-Cresyl glucuronide (µmol/L)	0.52 ± 0.14		47 ± 64
p-Cresyl sulfate (µmol/L)	37 ± 18		985 ± 843
Hippuric acid (µmol/L)	43 ± 20		817 ± 931
Cystatin C (mg/L)	0.13 ± 0.05		0.22 ± 0.08
GFR (mL/min)	58 ± 18	22 ± 14	
ERBF (mL/min)	1409 ± 325	412 ± 210	

Mean (±SD) plasma concentrations of urea, creatinine and phosphate, glomerular filtration rate (GFR) and effective renal blood flow (ERBF) before and 1.5 ± 0.7 months following embolization (*n* = 1 measurement in *n* = 5 goats), and peak plasma concentrations during acute-on-chronic kidney injury (AKI), following gentamicin administration (10 mg/kg twice daily for 7–10 days) (*n* = 11 courses in *n* = 5 goats, 1–3 gentamicin courses per goat) or prior to termination.

**Table 2:** Results of the standard peritoneal permeability analysis in goats (*n* = 2).

SPA	MTAC Urea (mL/min)	MTAC Creatinine (mL/min)	MTAC Phosphate (mL/min)	D/P Urea t = 4 h	D/P Creatinine t = 4 h
1	3.8	2.3	3.0	0.4	0.3
2	6.4	5.1	6.3	0.4	0.3
3	7.9	6.0	4.8	0.6	0.5
4	7.2	5.5	5.8	0.7	0.5
Mean ± SD	6.3 ± 1.5	4.7 ± 1.4	5.0 ± 1.2	0.5 ± 0.1	0.4 ± 0.1

SPA, standard peritoneal permeability analysis; MTAC, mass transfer area coefficient; D/P, dialysate-to-plasma concentration gradient after a 4-h dwell.

## Data Availability

Not applicable.

## Supplementary Materials

The following are available online at https://www.mdpi.com/article/10.3390/biology10040292/s1, Figure S1: Creatinine plasma concentrations (µmol/L) during follow-up in *n* = 5 goats, excluding episodes of gentamicin-induced acute-on-chronic kidney injury. †In one goat, plasma creatinine stabilized at ~350 µmol/L after a severe episode of gentamicin induced acute-on-chronic kidney injury. *Number of goats in study. Figure S2. Creatinine plasma concentration-time profiles of *n* = 2 goats who were euthanized after 15 and 27 months of follow-up (A and B, resp.) because of unexplained severe acute-on-chronic kidney injury, which was not induced by gentamicin administration and did not recover spontaneously. a, first partial embolization; b, second partial embolization; c, gentamicin (10 mg/kg twice daily for 7 days) induced severe acute-on-chronic kidney requiring three intermittent HD sessions in five days; d, acute-on-chronic kidney injury treated with five intermittent HD sessions in nine days, followed by euthanasia in the absence of spontaneous recovery of kidney function; e, partial embolization; f-g, gentamicin (10 mg/kg twice daily for 7 days); h, euthanasia because of severe acute-on-chronic kidney injury. Figure S3. Activity of the classical (CH50) and alternative complement pathway (AP50). Complement-dependent lysis of erythrocytes was calculated as the percentage lysis relative to cells lysed in water (100% lysis) and cells incubated in buffer (0% lysis). Percentage lysis was plotted against serum dilution. The mean ± standard deviation of three serum samples of two goats is presented. Table S1. Gentamicin administration without an effect on urea, creatinine, phosphate and potassium plasma concentrations in goats (*n* = 2).
