# Supplementary material for: A Uremic Goat Model Created by Subtotal Renal Artery Embolization and Gentamicin
Source: Biology (Basel). 2021 Apr 3;10(4):292. doi: 10.3390/biology10040292 (PMC8066079; doi:10.3390/biology10040292)
Supplement: Supplementary file 1 [file biology-10-00292-s001.pdf]

biology

MDPI

Article

A Uremic Goat Model Created by Subtotal Renal Artery Embolization and Gentamicin

Maaike K. van Gelder, Joost C. de Vries, Sabbir Ahmed, Anneke S. Monninkhof, Gérard A.P. de Kort, Evert-Jan P.A. Vonken, Diënty H.M. Hazenbrink, Koen R.D. Vaessen, Tri Q. Nguyen, Marianne C. Verhaar, Jaap A. Joles and Karin G.F. Gerritsen

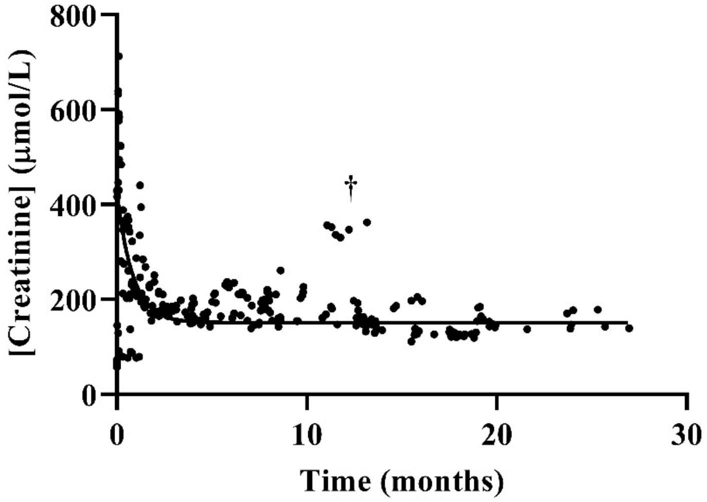

Month	0	9	13	16	27
N=*	5	4	3	1	0

Figure S1. Creatinine plasma concentrations (μmol/L) during follow-up in n = 5 goats, excluding episodes of gentamicin-induced acute-on-chronic kidney injury. †In one goat, plasma creatinine stabilized at ~350 μmol/L after a severe episode of gentamicin induced acute-on-chronic kidney injury. *Number of goats in study.

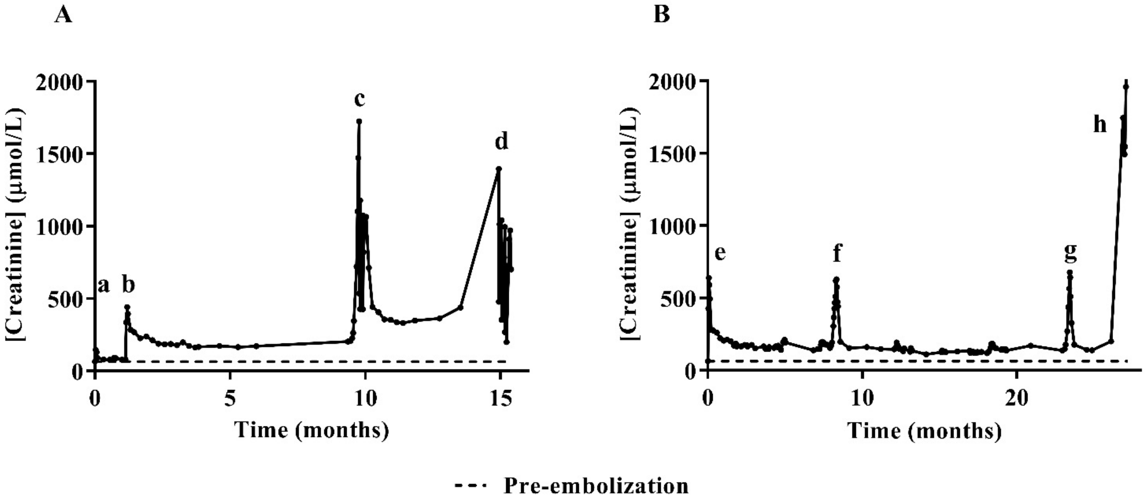

Figure S2. Creatinine plasma concentration-time profiles of n = 2 goats who were euthanized after 15 and 27 months of follow-up (A and B, resp.) because of unexplained severe acute-on-chronic kidney injury, which was not induced by gentamicin administration and did not recover spontaneously. a, first partial embolization; b, second partial embolization; c, gentamicin (10 mg/kg twice daily for 7 days) induced severe acute-on-chronic kidney requiring three intermittent HD sessions in five days; d, acute-on-chronic kidney injury treated with five intermittent HD sessions in nine days, followed by euthanasia in the absence of spontaneous recovery of kidney function; e, partial embolization; f-g, gentamicin (10 mg/kg twice daily for 7 days); h, euthanasia because of severe acute-on-chronic kidney injury.

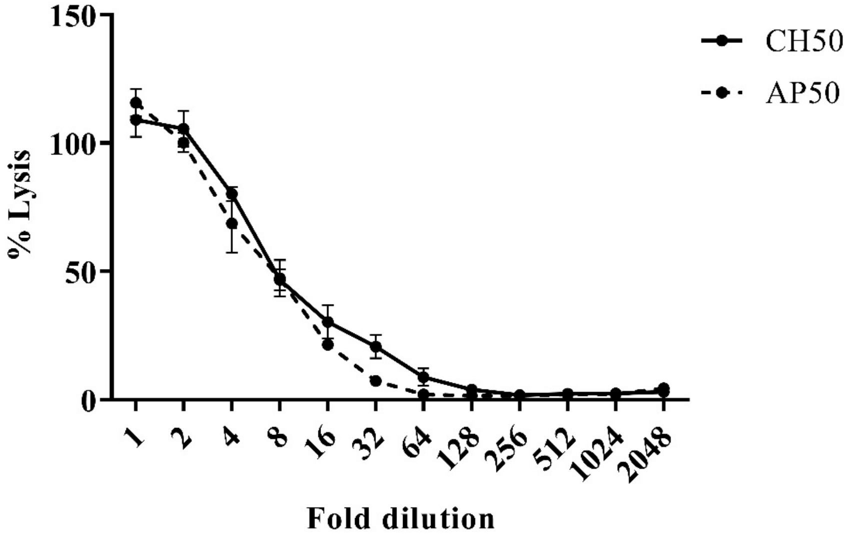

Figure S3. Activity of the classical (CH50) and alternative complement pathway (AP50). Complement-dependent lysis of erythrocytes was calculated as the percentage lysis relative to cells lysed in water (100% lysis) and cells incubated in buffer (0% lysis). Percentage lysis was plotted against serum dilution. The mean ± standard deviation of three serum samples of two goats is presented.

Table S1. Gentamicin administration without an effect on urea, creatinine, phosphate and potassium plasma concentrations in goats (n = 2).

Goat	Gentamicin dose	Time (days)*	Time C_{max} (days)†	Urea (mmol/L)		Creatinine (µmol/L)		Phosphate (mmol/L)		Potassium (mmol/L)	
				C_{pre}	C_{max}	C_{pre}	C_{max}	C_{pre}	C_{max}	C_{pre}	C_{max}
1	5 mg/kg 2d1	5	9	14.9	14.0	191	220	1.66	2.12	3.9	4.8
2	5 mg/kg 2d1	5	-	15.0	13.7	165	160	1.39	-	4.4	5.1
1	10 mg/kg 2d1	4	5	14.3	14.4	210	266	1.75	2.87	4.6	4.4
2	10 mg/kg 2d1	4	9	11.4	10.4	156	171	1.21	-	4.3	4.0
2	10 mg/kg 2d1	6	9	12.8	13.1	142	213	1.26	-	3.8	3.9
2	10 mg/kg 2d1	6	7	10.1	17.1	145	177	1.56	1.40	4.3	5.1
2	10 mg/kg 2d1	7	12	11.4	13.8	119	184	1.79	1.75	4.1	5.1
2	10 mg/kg 2d1	8	11	5.0	13.9	147	198	1.61	1.70	4.6	4.8

*Duration of gentamicin administration. †Time C_{max}, time to achieve maximum plasma concentration. ‡C_{pre} *vs* C_{max} analyzed with a Wilcoxon matched-pairs signed rank test. C_{pre}, plasma concentration at day 1 prior to gentamicin administration; C_{max}, maximum plasma concentration after gentamicin administration.
